# Early-Life Gut Health Indicators and Reported Prevalence of Infant Functional Constipation by Healthcare Professionals

**DOI:** 10.3390/nu15020298

**Published:** 2023-01-06

**Authors:** Leilani Muhardi, Dianne J. M. Delsing, Irina Zakharova, Koen Huysentruyt, Sze-Yee Chong, Ruey Terng Ng, Andy Darma, Badriul Hegar, Mohammed Hasosah, Erick Toro-Monjaraz, Merih Cetinkaya, Chung-Mo Chow, Marion M. Aw, Urszula Kudla, Louise Naz West, Yvan Vandenplas

**Affiliations:** 1 FrieslandCampina AMEA, Singapore 039190, Singapore; 2 FrieslandCampina, 3818 LE Amersfoort, The Netherlands; 3 Department of Pediatrics, Russian Medical Academy Continuous Professional Education of the Ministry of Health of Russian Federation, Moscow 125993, Russia; 4 KidZ Health Castle, Department of Pediatric Gastroenterology, UZ Brussel, Vrije Universiteit Brussel (VUB), 1090 Brussels, Belgium; 5 Department of Pediatrics, Hospital Raja Permaisuri Bainun, Ipoh 30450, Malaysia; 6 Department of Pediatrics, University of Malaya, Kuala Lumpur 50603, Malaysia; 7 Department of Pediatrics, Faculty of Medicine, Universitas Airlangga, Surabaya 60131, Indonesia; 8 Department of Pediatrics, Faculty of Medicine, Universitas Indonesia, Jakarta 10430, Indonesia; 9 Department of Pediatric, King Saud Bin Abdulaziz University for Health Sciences, Jeddah 14611, Saudi Arabia; 10 King Abdullah International Medical Research Center (KAIMRC), Jeddah 11481, Saudi Arabia; 11 Unit of Physiology and Gastrointestinal Motility, Gastroenterology and Nutrition Department, National Institute of Pediatrics, Mexico City 04530, Mexico; 12 Department of Neonatology, Health Sciences University, Basaksehir Cam and Sakura City Hospital, Istanbul 34480, Turkey; 13 Virtus Medical Group, Hong Kong; 14 Department of Paediatrics, Yong Loo Lin School of Medicine, National University of Singapore, Singapore 119077, Singapore; 15 Department of Paediatrics, Khoo Teck Puat-National University Children’s Medical Institute, National University Health System, Singapore 119228, Singapore

**Keywords:** gut health indicators, infant, toddlers, healthcare professionals, e-survey

## Abstract

A healthy gut during early childhood is important. However, it seems that there are no standard indicators used to assess it. Healthcare professionals (HCPs) were asked via an electronic survey question about gut health indicators (GHIs) for infants and toddlers, in addition to an estimated prevalence of infant’s functional constipation (FC) and its management. HCPs from eight countries participated in the survey (Russia (66.0%, 1449), Indonesia (11.0%, 242), Malaysia (6.0%, 132), Mexico (5.7%, 125), KSA (5.1%, 113), Turkey (3.0%, 66), Hong Kong (2.2%, 49), and Singapore (1.0%, 23)). The 2199 participating respondents were further classified into three continents (Asia (20.2%), Europe (68.8%), and others (11.0%)). Most of them were pediatricians (80.3%), followed by pediatric gastroenterologists (7.0%), general practitioners (6.4%), and others (6.3%). The top three preferred GHIs were similar for infants and toddlers: an absence of gastrointestinal (GI) symptoms, effective digestion/absorption as assessed by normal growth, and a general feeling of well-being. The absence of GI-related infection was the least preferred indicator. Most of the respondents reported the prevalence of FC among infants was less than 5%, with the peak incidence between the ages of 3 and 6 months. The reported choices of intervention to manage FC in infants were a change to a specific nutritional solution from a standard formula (40.2%), parental reassurance (31.7%), and lactulose (17.0%). Conclusion: The HCPs in the eight countries preferred the absence of GI symptoms, normal growth for effective digestion and absorption, and general well-being as the gut health indicators in infants and toddlers. The reported prevalence of FC in infants was less than 5%.

## 1. Introduction

Infants undergo rapid organ development, including the gastrointestinal (GI) tract, during the first months of life. The stomach volume capacity of an infant is only about 10% of that of an adult [1]. The activity of various digestive enzymes, especially lipase and protease, is quite limited during the first few months of life in comparison to the levels in adulthood [2,3]. In addition, the GI tract motility and its functionality will only mature over time [4].

The determination of markers indicating gut health is relevant to track the development of GI tract functionality. Different indicators for a healthy gut in adults have been proposed, which includes efficient absorption and digestion, a stable and balanced gut microbiota, an absence of GI disease, a balanced GI immune system, and an overall feeling of well-being [5].

Some of the above-mentioned indicators could also be applicable to young children. An effective GI absorption and digestion of nutrients in young children will result in normal nutritional status and normal growth [5]. An absence of gastro-esophageal reflux (disease), constipation, celiac disease, and many other GI illnesses indicates, of course, the importance of an “absence of disease” when describing gut health [5]. In early life, functional gastrointestinal disorders (FGIDs), which consist mainly of gastroesophageal reflux/regurgitation, colic/excessive crying/irritability, and constipation, are commonly reported [6]. As the large intestine of an adult harbors more than 10^14^ bacteria, an optimal gut microbiota or an absence of dysbiosis is also an important aspect to consider when determining gut health also in children [7]. This microbiota in combination with the GI barrier function contributes to the overall immune system, GI tract function, and general feelings of well-being through the gut–brain communication [5]. Therefore, overall well-being could also be part of the gut health indicators. This could be indicated by a normal quality of life [5] in adulthood, a happy and smiling baby [8], or a long night’s sleep in young infants [9].

There are no guidelines or recommendations for healthcare professionals (HCPs) on how to assess a healthy gut during infancy and toddlerhood. Therefore, this survey aims to understand the perspective of HCPs on (i) the preferred gut health indicators and (ii) the estimated prevalence of FC during infancy.

## 2. Methodology

An international survey was performed in 8 countries, as described in a previous publication [10]. Pediatricians, pediatric gastroenterologists, and general practitioners from eight countries/regions (Hong Kong, Indonesia, Kingdom of Saudi Arabia, Malaysia, Mexico, Russia, Singapore, and Turkey) participated anonymously via the SurveyMonkey. The electronic survey was implemented between August 2021 and March 2022 by sending the link to the principal investigators who forwarded this link randomly via email or WhatsApp platform to local respondents, who were part of the network of the principal investigator. The countries were selected based on the initiators of the study’s network (authors L.M. and Y.V.) and then divided into three regions based on geographical areas whenever possible, namely Region 1 (Asia: consisting of Indonesia, Malaysia, Hong Kong, and Singapore), Region 2 (Europe, consisting of Russia and Turkey), and Region 3 (Rest of the world, including KSA and Mexico).

Ethics approval was obtained from each participating center before the dissemination of the survey (i.e., the Independent Interdisciplinary Committee on Ethical Review of Clinical Trials in Russia, the University Medical Research Ethics Committee of the University of Malaya in Malaysia, the Ethics Committee of the Airlangga University in Indonesia, King Abdullah International Medical Research Center in Kingdom of Saudi Arabia, Instituto Nacional de Pediatria in Mexico, Basaksehir Cam ve Sakura Sehir Hastanesi in Turkey, Hong Kong Doctors Union, National University Singapore). Filling out the questionnaire was regarded as having provided consent to participation as per the explanation in the introduction section.

There were 30–40 questions in the survey, which consisted of, among others, demographic questions, FC-related questions, including the diagnostic criteria for, the prevalence of, and the management of FC, and gut health indicators. All questions were close-ended with a multiple-choice format. In this current paper, the answers to the questions (*n* = 2) on infant/toddler gut health indicators and FC in infants (*n* = 6) are addressed.

The HCPs were asked to select their preferred indicators of gut health in infants/toddlers from the following list:

i. Absence of GI discomfort symptoms, i.e., no FC;
ii. Absence of GI-related infection;
iii. Effective digestion and absorption of food as indicated by normal growth;
iv. Well-being status, i.e., no excessive crying, good sleep during the night, good quality of life of the parents, and stool consistency and frequency;
v. Strong immune function due to an optimal gut microbiota diversity.

An example of the full questionnaire has been previously published [9]. The questions addressed in the current paper, including all response options, can be found in Appendix A.

The statistical analyses were conducted using the IBM SPSS System for Windows, version 24 (IBM Corp., Armonk, NY, USA) with the following exclusion criteria: (1) no constipation cases were observed by the respondent in his/her practice in the last week, and (2) less than 50% of the questions were answered. The qualitative data in the descriptive statistics are presented as absolute numbers and percentages (nominator: the number for each response, and denominator: the total number of responses) to report the difference in the actual responses. Each question can have a different denominator depending on the number of responses and the missing values. The differences between categories (type of profession, years of practice, and region) were assessed using the chi-squared test or Fisher’s exact test. A statistically significant difference is considered when the *p*-value is <0.05.

## 3. Results

### 3.1. The Demographics of Respondents

**Figure 1:**
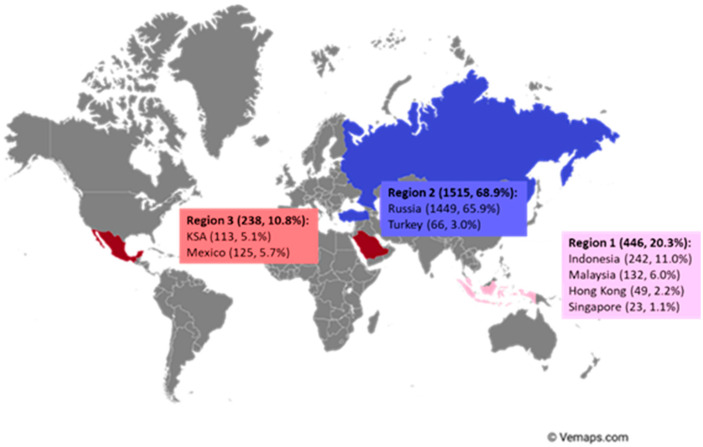
The geographical location of the survey respondents (number of respondents and % of total respondents).

A total of 2199 (85%) out of 2596 healthcare professionals were included in the data analysis as there were incomplete responses (see Figure 1).

**Figure 2:**
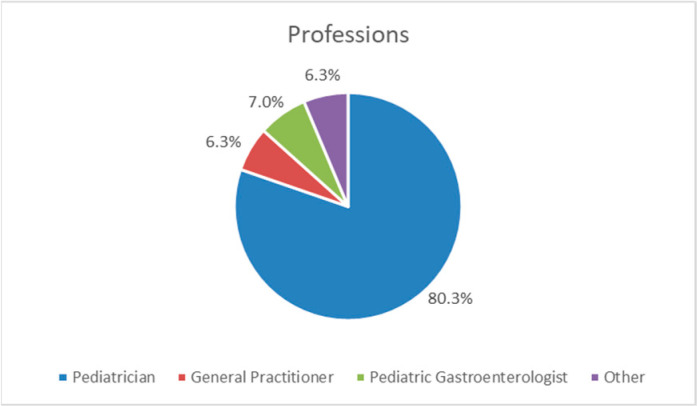
The professions of the respondents as a percentage of the total number of respondents.

The majority of respondents (80.3%, 1759/2191) were pediatricians, followed by pediatric gastroenterologists (7%, 154/2191), general practitioners (6.4%, 139/2191), and others (6.3%, 139/2191) which included residents (Figure 2).

#### 3.1.1. Indicators of Gut Health in Infants and Toddlers

The answers regarding gut health indicators from 2143 respondents (97.4%) were analyzed. In Singapore, the respondents were asked to select the top three gut health indicators without providing any ranking, while in other countries, the respondents were asked to rank the three most important gut health indicators. As a consequence, the data from Singapore could not be included in the analysis.

**Figure 3:**
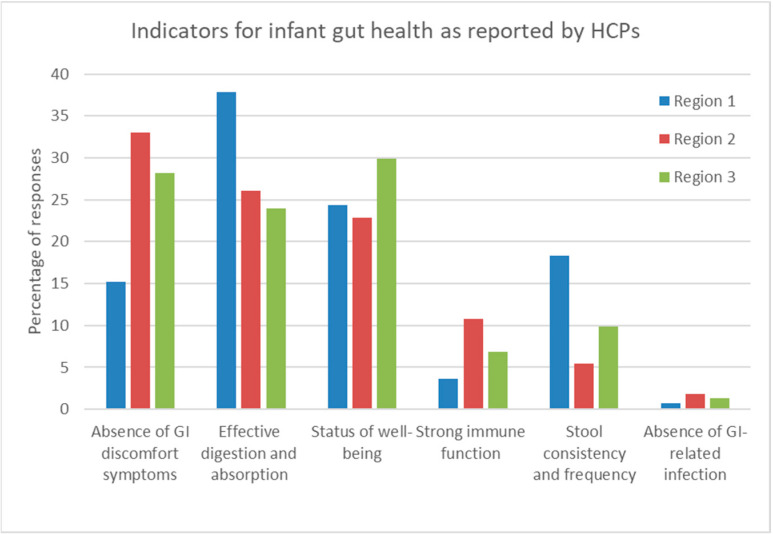
Percentages of responses by HCPs regarding their preferred indicators of infant gut health per region. Region 1 (Asia: consisting of Indonesia, Malaysia, and Hong Kong), Region 2 (Europe, consisting of Russia and Turkey), and Region 3 (Rest of the world, including KSA and Mexico).

The following three indicators for infant gut health were ranked the highest: (i) “absence of GI-discomfort symptoms” (622/2143, 29.0%); (ii) “effective digestion and absorption of food as indicated by normal growth” (603/2143, 28.1%); and (iii) “feeling of well-being” (513/2143, 23.9%) (Figure 3). The lowest score was for “the absence of GI-related infection” (33/2143, 1.4%). However, the indicator “stool consistency and frequency” scored in the top three in Region 1 (Asia) but not in the other regions (*p* < 0.01).

**Figure 4:**
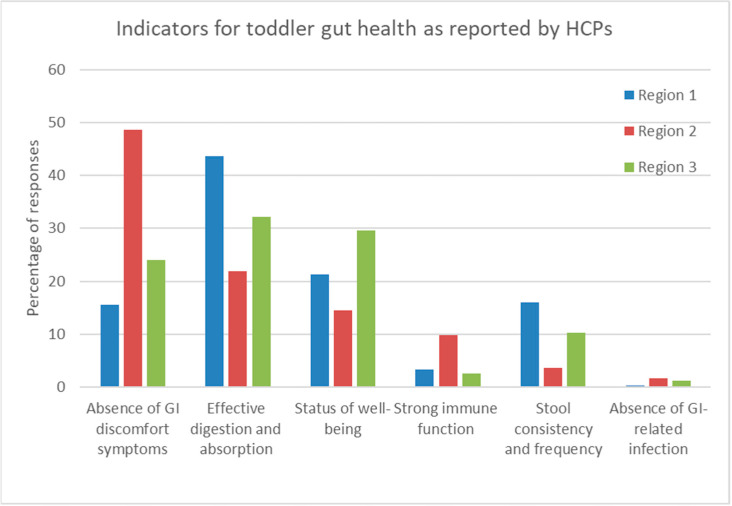
Percentages of responses by HCPs regarding their preferred indicators of toddler gut health per region. Region 1 (Asia: consisting of Indonesia, Malaysia, and Hong Kong), Region 2 (Europe, consisting of Russia and Turkey), and Region 3 (Rest of the world, including KSA and Mexico).

Regarding gut health in toddlers, the HCPs selected the same top three indicators as for infants: (i) “absence of GI-discomfort symptoms” (842/2131, 39.5%); (ii) “effective digestion and absorption of food as indicated by normal growth” (580/2131, 27.2%); and (iii) “feeling of well-being” (372/2131, 17.5%). Figure 4 represents the preferred toddler gut health indicators per region. Again, “stool consistency and frequency” was within the top three gut health indicators for toddlers in Region 1 (Asia) but not in the other regions (*p* < 0.01).

#### 3.1.2. Prevalence of FC in Infants

**Figure 5:**
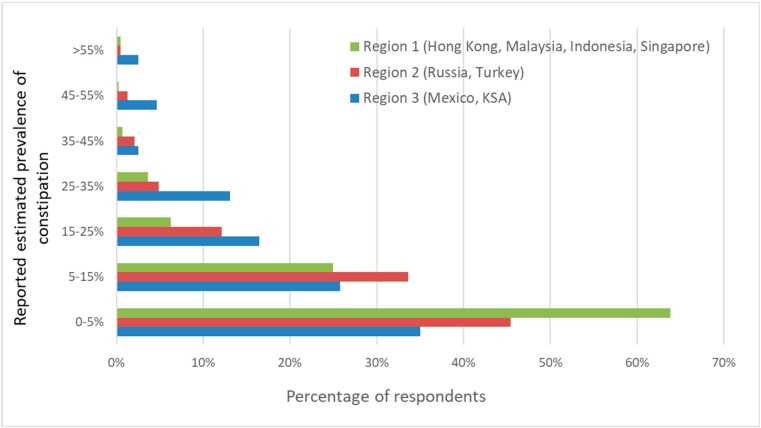
The estimated reported prevalence of FC in infants by region (in %). Overall, there are significant differences in the distribution of estimated prevalence between regions (*p* < 0.001).

The estimated percentage of constipation cases was significantly different across the regions. In Region 1, representing Asian countries, a statistically significantly higher percentage of HCPs reported a prevalence of FC of less than <5% compared to the other regions (Region 1: 248/445, 63.8% vs. Regions 2 and 3: 685/1507, 45.5% and 83/237, 35%, respectively (*p* < 0.001)) (Figure 5). According to the responses of the HCPs, the prevalence of FC is generally estimated to be lower in Asian countries than in the other regions. Furthermore, more HCPs in Region 3 estimated the prevalence of FC to be above 55% than in the other regions: 2.5% (6/237) versus 0.4% (2/445) in Region 1 and 0.5% (7/1507) in Region 3.

Most HCPs reported that, according to their experience, the peak age of FC in infants is between 3 and 6 months (761/2191, 34.7%), in comparison to 0–3 months, 6–9 months, or 9–12 months.

#### 3.1.3. Impact of FC in Infants on Quality of Life

The respondents were asked to rate their observations on the influence of an infant’s FC on the quality of life (QoL) of the family. The following options were proposed: “almost always” (in more than >70% of the cases), “sometimes” (between 30 and 70% of the cases), “rarely” (between 10 and 30% of the cases), or “never” (in less than 10% of the cases).

The most reported frequency of FC affecting QoL was “sometimes” (in 30–70% of cases) (754/2182, 34.6% of all responses), with a significant difference between professions (*p* < 0.001) (Table 1). Pediatric gastroenterologists were more likely to report the observation of FC affecting QoL (“almost always”; >70% of cases) (84/154, 54.5%), whereas the other HCPs were more likely to answer that they observed it sometimes (30–70% of cases). General practitioners more frequently replied that, according to their observations during practice, FC does not affect QoL (22/138, 15.9%).

#### 3.1.4. Management of FC in Infants

**Figure 6:**
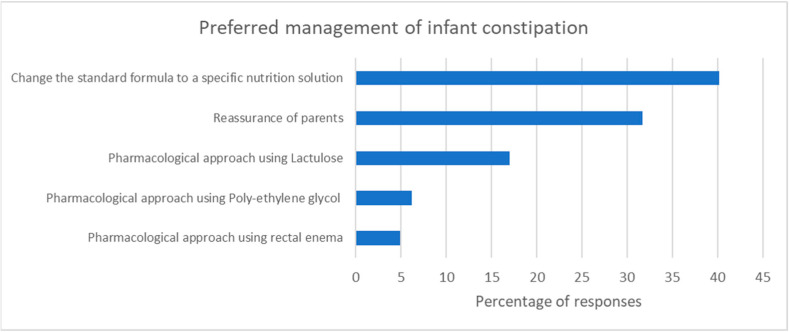
The reported first choice for the management of FC in infants (% of total number of respondents).

The HCPs preferred the change from a standard formula to a specific nutritional solution to manage FC in infants (40.2%, 835/2077), parental reassurance (31.7%, 659/2077), and the use of lactulose (17.0%, 353/2077) (Figure 6). More experienced HCPs (>10 years) reported they preferred to use a particular specific nutritional solution, while less experienced HCPs preferred to opt first for parental reassurance to manage infantile FC (*p* < 0.001).

#### 3.1.5. Preferred Nutritional Management of FC in Infants

**Figure 7:**
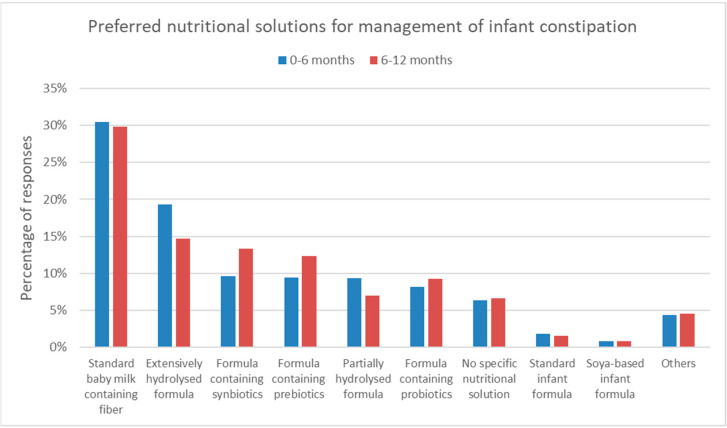
The preferences of HCPs for nutritional solutions in the management of FC in infants.

The questionnaire asked the HCPs to select their preferred nutritional solution for the management of FC in infants between 0 and 6 months or between 6 and 12 months of age. The top three preferred options were identical for infants of 0–6 months and of 6–12 months. The preferred option was a standard formula containing fiber (667/2186, 31.5% and 625/2095, 29.8%), followed by an extensively hydrolyzed formula (423/2186, 19.4% and 308/2095, 14.7%). The third most preferred nutritional solution was a formula containing synbiotics (211/2186, 9.7% and 280/2095, 13.4%) (Figure 7).

## 4. Discussion

Digestion and absorption of nutrients resulting in optimal growth are the most important functions of the GI tract. The younger an infant is, the more important adequate growth is [11]. As a consequence, it is quite understandable that around 30% of HCPs selected “effective digestion and absorption of food as indicated by normal growth” as the most important determinator of healthy gut function in young children. Disturbed or partial digestion and absorption of nutrients, e.g., of dietary protein or fat, are a risk for sub-optimal growth [2,12]. Thus, optimal growth can be considered as a proxy for adequate digestion and absorption. In contrast, rapid weight gain resulting in being overweight cannot be used as a marker of efficient absorption and digestion functions, although it is a consequence of an imbalance between caloric intake and energy expenditure [13]. Therefore, when defining optimal growth, body weight and height are not always the best parameters, and other variables, such as body composition, should be considered even in early life [14].

According to the participating HCPs, the second-best indicator for infant gut health was the ‘absence of GI symptoms related to functional GI disorders (FGIDs)’. FGIDs are commonly reported to occur during early life [15,16,17]. According to the literature, the prevalence is between 20 and 50% in unselected populations of infants, and many infants present with a combination of different FGIDs [18]. According to age, FGIDs usually start with regurgitation, irritability, and colic in the earlier months of life and continue with functional constipation (FC) in older infants and toddlers [19]. For the current survey, only information on FC in young children was obtained.

The majority of the respondents estimated that the prevalence of FC was below 5%, especially in the Asian region This corresponds with the relatively low FC prevalence reported previously for China and Malaysia: 1.5% and 1.3%, respectively [17,20]. This low prevalence in the Asia region may be explained by the fact that stool consistency and frequency are one of the top indicators for gut health chosen by the HCPs in this region. It is part of the cultural belief of some of the ethnic groups in Asia that passing daily stools with soft consistency is an important indicator of health. Every necessary means will be undertaken to ensure good bowel movements and, hence, the low prevalence of reported FC in this region.

For the other regions, the estimated prevalence of constipation was almost equally distributed over “less than 5%”, “between 5 and 15%”, and “between 15 and 25%”. These data suggest a higher prevalence of infant constipation in non-Asian countries. Indeed, a previous review reported an FC prevalence between 3 and 17.7% in different countries outside Asia (USA, Turkey, African countries, Jordan, The Netherlands, Belgium, and Italy) [6]. The prevalence of FC in young children in Hong Kong, Indonesia, Mexico, Russia, and Singapore has not been previously reported. For these countries, the prevalence of FC in infants in our study can be considered an indicator of primary FC prevalence.

In this survey, the peak of FC in infancy was reported to be at 3–6 months of life. Switching from breastfeeding to formula feeding or introducing solid meals to an infant’s diet is widely noted as a cause of FC [21]. This may explain the reported peak in prevalence at 3–6 months in infants, although it needs to be noted that data on feeding practices were not collected in the current survey. Although plausible, not all studies find a difference in FC prevalence over the first year of life; an earlier study in China reported a similar incidence of FC at 0–6 months and 6–12 months [17].

FC can cause emotional and physical distress for the affected infants and their parents, which could lower the family’s quality of life (QoL) [22]. The children deal with physical discomfort, which can lead to excessive irritability and distress, increasing both the children’s and the parents’ stress levels [23]. Most of the respondents in this survey reported FC affecting the quality of life in 30–70% of cases. The main reason put forward was pain during defecation, followed by parental stress and excessive crying. This indicates that proper management of FC in infants can have an important impact on the QoL of the entire family. Interestingly, in the current survey, pediatric gastroenterologists reported FC affecting QoL more often than other HCPs. This may reflect the fact that pediatric gastroenterologists see the more severe cases, or that they are better trained to diagnose and manage FC compared to other groups of HCPs [24]. Pediatric gastroenterologists who are usually placed in tertiary centers see more severely affected and complex cases. Differences in the perception of QoL among various ethnic groups have also been previously reported [25].

The recommendation for FC management includes adequate intake of fibers and fluids and physical activity. Pharmacological treatment is only warranted in severe cases [26]. Furthermore, educating and reassuring parents remain important, with the goal of restoring harmony by reducing infant stress and relieving parental anxiety [27]. In addition, the early onset of FC management has been suggested to contribute to its resolution and, thus, reduced the risk of chronic constipation [18]. In this survey, the HCPs’ preferred nutritional managements for FC were (1) changing to a specific nutrition solution from a standard formula, (2) parental reassurance, and (3) pharmacological treatment. More experienced HCPs preferred to offer a nutritional solution to parents rather than just reassurance. This could be due to their experience in understanding the psychological needs of parents and in avoiding subsequent doctor hopping.

Nutritional solutions could include infant formula products containing supplementation with fibers, such as galacto-oligosaccharides (GOS) and/or fructo-oligosaccharides (FOS), probiotics, or a different lipid source than vegetable oils. Multiple studies have shown that supplementation of infant formula with GOS and/or FOS does soften stools and increase stool frequency [28]. In addition, harder stools are frequent in infants fed with formulas containing vegetable oil rich in palmitate in the stereospecific numbering (Sn) positions Sn-1 and Sn-3, favoring the formation of calcium soaps [29]. In human milk, palmitic acid is mainly in the Sn-2 position, which is in contrast with those in bovine milk [30]. The palmitic acid in the Sn-1 and Sn-3 positions favor the formation of calcium soaps which could lead to hard(er) stools. In contrast, the palmitic acid in the Sn-2 position is not associated with this problem. Recent studies have shown that infants have softer stools and reduced fecal palmitate soaps upon consumption of bovine milk containing natural milk fat rich in PA in the sn-2 position compared to a vegetable fat-based formula [31]. In addition, the application of structured triglycerides with a high sn2-palmitic acid content (OPO) in infant formula has been shown to reduce calcium soaps in feces and enhance stool frequency [32].

To our knowledge, the current study is the first to describe the most common way for HCPs to define a healthy gut in infancy and childhood. Nonetheless, there are a few limitations, as previously described [10]. Even though the survey has more than two thousand participants, the number of respondents from each country is unequal among the participating countries. In addition, the percentage of respondents toward the total number of HCPs differs from one country to another due to the total number of HCPs in each country. There could be a selection bias in this survey due to (1) the limited distribution of the survey to those within the specific network of the investigators, and (2) those who have experience with online surveys might be more likely to respond to the survey. The data are dependent on the respondents’ opinions, which could limit the survey’s objectiveness. In addition, we only investigated one aspect of FGIDs, i.e., FC, in-depth. Additional questions on other FGID symptoms, such as reflux and colic, may have given a more complete picture of issues impacting early-life gut health.

On the other hand, the current study gives important insights into how HCPs assess a healthy GI function in infants and toddlers. These indicators can be also communicated to parents. In addition, this survey adds valuable and reliable complementary information on FC in infants at a time when primary data collection was difficult due to the COVID-19 pandemic.

## 5. Conclusions

An absence of GI symptoms and normal growth as a proxy for absorption and digestion are the two top indicators for infant gut health according to the opinion of HCPs in eight countries. One of the GI symptoms that infants may suffer from is FC, and it is mostly reported in this survey with a prevalence of less than 5% among infants. The switch to a specific nutritional solution from standard infant formula and parental reassurance are the preferred reported methods for FC management in infants. Normalization of stool frequency and consistency can have a positive and meaningful impact on improving the QoL of infants and their families.

## Figures and Tables

**Table 1:** The frequency of HCPs by profession reporting to have observed FC affecting (QoL) of infants and their parents in the last week.

Frequency of Observing FC Affecting QoL ^#^	Overall	Pediatricians	General Practitioners	Pediatric Gastro-enterologists	Other Professions
Almost always (>70%)	703/2182 (32.2%)	539/1751 (30.8%)	36/138 (26.1%)	**84/154** **(54.5%)**	44/139 (31.7%)
Sometimes (30–70%)	**754/2182 (34.6%)**	**618/1751 (35.3%)**	**41/138** **(29.7%)**	46/154 (29.9%)	**49/139** **(35.3%)**
Rarely (10–30%)	575/2184 (26.4%)	481/1751 (27.5%)	39/138 (28.3%)	21/154 (13.6%)	34/139 (24.5%)
Never (<10%)	150/2184 (6.9%)	113/1751 (6.5%)	22/138 (15.9%)	3/154 (1.9%)	12/139 (8.6%)

Data are presented as the number of responses/total responses (percentage of total responses). Results in **bold** represent the highest response. # *p* < 0.001 between professions.

## Data Availability

Data is available upon request.

## Appendix A

Example of the questions in the survey relevant for the information in the manuscript.

1. In your opinion, what will be the best indicator to assess healthy GI tract in infants: 1. Absence of GI discomfort symptoms i.e., no constipation 2. Absence of GI-related infection 3. Effective digestion and absorption of food as indicated by normal growth 4. Status of well-being ie no excessive crying, good sleep during the night, good quality of life of the parents 5. Stool consistency and frequency 6. Strong immune function from inside due the optimal gut microbiota diversity 7. Others; please specify: _____________________
2. In your opinion, what will be the best indicator to assess healthy GI tract in toddlers: 1. Absence of GI discomfort symptoms i.e., no GER, no constipation + crying, no colic 2. Absence of GI-related infection 3. Effective digestion and absorption of food as indicated by normal growth 4. Status of well-being ie no excessive crying, good sleep during the night, good quality of life of the parents 5. Stool consistency and frequency 6. Strong immune function from inside due the optimal gut microbiota diversity 7. Others; please specify: _____________________
3. What is the average prevalence of infantile constipation in your practice within the last one week? 1. 0–5% 2. 6–15% 3. 16–25% 4. 26–35% 5. 36–45% 6. 46–55% 7. More than 55%
4. At which age do you encounter the highest incidence of the infant’s constipation in your practice? 1. 0–2.9 months 2. 3–5.9 months 3. 6–8.9 months 4. 9–12 months 5. Others: ___________________
5. How frequently do you observe constipation affects the quality of life of infants and their parents in the last week? 1. Almost Always: >70% 2. Sometimes: 30–69% 3. Rarely: 10–29% 4. Never: <10%
6. Please rank the following treatments that you usually practice when managing constipation in infants 0–12 months: (no#1: the most frequent; no#6: the least frequent) 1. Change the standard formula to a specific nutrition solution 2. Pharmacological approach using Lactulose 3. Pharmacological approach using Poly-ethylene Glycol (PEG) 4. Pharmacological approach using rectal enema 5. Pharmacological approach using Sodium picosulfate 6. Reassurance of parents
7. Which specific nutrition solution do you often use in managing constipation in nonbreastfed 0–6 months old infants? 1. Extensively hydrolysed formula 2. Formula containing fiber (i.e., inulin or carob bean gum) 3. Formula containing milk-fat 4. Formula containing prebiotics (fructo-oligosacharides or galacto-oligosaccharides) 5. Formula containing probiotics (*Bifidobacteria* or *Lactobacillus* Sp.) 6. Formula containing synbiotics (combination between prebiotics and probiotics) 7. Goat milk–based infant formula 8. Magnesium-rich formula 9. No specific nutrition solution 10. Partially hydrolyzed formula 11. Soya-based infant formula 12. Standard infant formula 13. Don’t know 14. Others; please specify: _____________________________
8. Which specific nutrition solution do you often use in managing constipation in nonbreastfed 6–12 months old infants? 1. Extensively hydrolysed formula 2. Formula containing fiber (i.e., inulin or carob bean gum) 3. Formula containing milk-fat 4. Formula containing prebiotics (fructo-oligosacharides or galacto-oligosaccharides) 5. Formula containing probiotics (*Bifidobacteria* or *Lactobacillus* Sp.) 6. Formula containing synbiotics (combination between prebiotics and probiotics) 7. Goat milk–based infant formula 8. Magnesium-rich formula 9. No specific nutrition solution 10. Partially hydrolyzed formula 11. Soya-based infant formula 12. Standard infant formula 13. Don’t know 14. Others; please specify: _____________________________
